# Impact of Stress on Health and Final Weight in Fattening Lambs

**DOI:** 10.3390/ani10081274

**Published:** 2020-07-25

**Authors:** Teresa Navarro, José María González, Juan José Ramos, María Carmen Marca, Lucia Figliola, Marta Ruiz de Arcaute, Marta Borobia, Aurora Ortín

**Affiliations:** 1 Departamento de Patología Animal, Instituto Agroalimentario de Aragón-IA2 (Universidad de Zaragoza-CITA), Facultad de Veterinaria, C/Miguel Servet 177, 50013 Zaragoza, Spain; teresanarr@gmail.com (T.N.); jmgsovino@gmail.com (J.M.G.); jjramos@unizar.es (J.J.R.); cmarca@unizar.es (M.C.M.); lucia.figliola@unifg.it (L.F.); martarda@unizar.es (M.R.d.A.); mborobia@unizar.es (M.B.); 2 Department of the Sciences of Agriculture, Food and Environment (SAFE), University of Foggia, Via Napoli 25, 71122 Foggia, Italy

**Keywords:** fattening lambs, feedlots, stress, health

## Abstract

**Simple Summary:**

In recent years, the lamb production system in North-Eastern Spain has undergone an important change and the number of lamb feedlots has increased significantly. However, the possible impact of stressors associated with this production system on the health and productive performance of the lambs has not been investigated. In this study, the stress level of Rasa Aragonesa fattening lambs, finished at a commercial feedlot or on the farm of birth, was measured throughout the fattening period. Our results show that, although feedlot lambs seemed to have experienced higher stress levels than those fattened on the farm of origin, finishing location did not affect the probability of presenting clinical signs of illness and ovine respiratory complex lesions, or the final weight of the lambs. Notwithstanding, associations were found between the concentration of fecal cortisol metabolites (a chronic stress indicator) at the time of weaning and the health condition and the final weight of the lambs, regardless of the location at which they were fattened. The stress level experienced by lambs prior to finishing seems to be relevant for their health and productive performance, and measures to reduce this stress should be further investigated as a useful tool to improve the sustainability of the ovine production system.

**Abstract:**

In order to determine whether the stress level had any influence on the health and final weight of Rasa Aragonesa fattening lambs, stress markers were measured throughout the fattening period in 80 feedlot lambs (group F) and in 40 lambs finished on the farm of birth (group C). The highest values of the stress indicators—neutrophil-to-lymphocyte-ratio (N/L), non-esterified fatty acids (NEFA), serum cortisol and fecal cortisol metabolites (FCM)—were recorded after the road transportation of group F to the feedlot. No differences between the groups were identified at the end of the adaptation period, but at the end of the fattening, statistically significant higher values for NEFA and serum cortisol were determined in group F. However, statistically significant differences between the groups were not found in the percentage of lambs with clinical signs of illness and ovine respiratory complex (ORC) lesions or in the final weight of the lambs. Independent of the location at which the lambs were fattened, those with FCM values at the beginning of the study (at the time of weaning) in the highest quartile developed a higher percentage of clinical signs of illness (45.5% vs. 32.1%, *p* > 0.05) and ORC lesions (45.5% vs. 10.7%, *p* < 0.01), and their average final weight was lower (24.36 kg vs. 27.40 kg, *p* = 0.001) than those with values in the lowest quartile. Stress experienced by lambs prior to finishing seems to be relevant for their further development, and FCM concentration at the time of weaning could be used as an indicator of health and productive performance of the lambs during the fattening period, regardless of finishing location.

## 1. Introduction

The sheep industry in North-Eastern Spain has experienced a significant change in recent years, and lamb production systems that include an intermediate step between the farm and the abattoir, at feedlots or classification centers (CC), are increasingly widespread [1]. These centers are utilized to standardize production and improve economic viability by reducing production costs and increasing sales revenue. However, this production system could have a negative impact on the welfare of the lambs. Stress factors related to management practices associated with this production system (transport to the feedlot, regrouping of animals of different origins, adaptation to a new environment) could contribute to increasing stress levels and compromise the function of the immune system, with detrimental effects on animal health [2]. Ovine respiratory complex (ORC) is the most important pathological process during this productive period in the Mediterranean area. ORC is caused by bacteria which are part of the microbiota of the respiratory system in healthy sheep [3], which are able to induce disease during periods of lowered immunological defense [1].

Several studies with lambs have analyzed the effect of transportation [4,5,6] and the stay at CC [7,8,9,10] on some stress markers. However, they did not address all stages of the process, nor did they introduce a control group of animals fattened by the traditional method, which could fail to identify critical points within this production system. Furthermore, as far as we know, the possible impact of stress on lamb health and productivity during the fattening period has not been investigated. In this work, we have studied the evolution of the stress level in lambs throughout the fattening period, from the farm of origin to the end of the fattening at a feedlot, comparing these data with those from lambs fattened on the farm where they were born. The stress markers analyzed were as follows: serum cortisol, fecal cortisol metabolites (FCM) and the serum acute phase proteins haptoglobin (HP) and serum amyloid A (SAA), as well as other hematological and biochemical parameters. In addition, with a view to identifying any possible impact of stress, any clinical signs of illness throughout the experimental period were monitored, live weights recorded and consolidated pneumonic lesions recognized at the abattoir. 

## 2. Materials and Methods 

This experiment was carried out in a lamb feedlot (Casa de Ganaderos de Zaragoza SCL) located in Aragón (North-Eastern Spain), in collaboration with the Veterinary Faculty of Zaragoza. The experimental protocol was approved by the Ethics Committee for Animal Experiments from the University of Zaragoza (reference PI 49/14). The care and use of animals were performed accordingly with the Spanish Policy for Animal Protection RD53/2013, which meets the European Union Directive 2010/63 on the protection of animals used for experimental and other scientific purposes.

### 2.1. Animals and Experimental Design

One hundred and twenty Rasa Aragonesa lambs from two farms associated with the feedlot were selected at random from the animals destined to be fattened throughout all seasons in one year (2016–2017), and they were divided into two groups: group F (*n* = 80), lambs fattened in the feedlot, and group C, the control group (*n* = 40), with lambs fattened on the farm of birth. Male lambs without clinical signs of disease, 40–50 days-old and 12–15 kg live weight, were selected on the farms of origin at the time of weaning, when they were abruptly separated from their mothers. During lactation, the lambs had been located indoors with their dams and habituated to human contact. Ten F lambs and five C lambs were selected seasonally from each farm, on the same day for both. Following selection, F lambs were transported to the feedlot (50 km away from each farm) by road (approximately 1-h journey), in a truck compliant with the welfare regulations for the transportation of lambs. On arrival, lambs were classified and grouped with other lambs from different farms in indoor pens at a density < 2 lambs/m^2^. Lambs of the control group were also housed indoors, on the farm of origin, in pens at the same density as in the feedlot, and without visual and auditory contact with their mothers. The same concentrate, straw and water were administered ad libitum to both groups, and straw bedding was used. Lambs were not castrated and were vaccinated against ORC, following the same protocol in both groups. At four time points throughout the experimental period, samples of blood (with Ethylenediaminetetraacetic—EDTA—and without anticoagulant) and feces were collected from the lambs by trained personnel. Blood samples were collected initially, by jugular venipuncture, followed by the fecal samples, which were evacuated from the rectum by hand. The first sampling (T0) was collected on the farm of origin, 15–30 min after abrupt mother separation. At this moment, lambs were selected and sampled according to a list of randomized numbers and F lambs loaded into the truck. The second sample (T1) was collected only from group F lambs immediately following unloading on their arrival at the feedlot. Samples were collected from both groups at a further two time points: the end of the adaptation period after two weeks of fattening (T2) and the end of the fattening period, four weeks later, prior to transportation to the abattoir (T3). Both on the farms and in the feedlot, samples were taken at the same time of day in order to avoid the influence of circadian rhythms. Once collected, they were kept refrigerated for conveyance to the laboratory. On the same day of collection, the EDTA blood samples were submitted to hematological analysis, the blood samples without anticoagulant were processed to obtain sera, aliquoted and stored at −20 °C until analysis, and fecal samples were lyophilized and stored until they were assessed. The lambs were examined daily by the same clinician throughout the study to identify any clinical signs of illness, and their weights were registered weekly. A post-mortem examination of the viscera was performed at the abattoir.

### 2.2. Hematological Parameters

An automatic hematological analyzer (Vet-ABC, DIVASA-FARMAVIC S.A., Barcelona, Spain), was used to measure red blood cell (RBC) count, hemoglobin (HGB), hematocrit and total leukocyte numbers. The differential leukocyte count was determined on Quick Panoptic-stained blood smears, by a trained operator blinded to treatment group, counting a minimum of 100 cells under a light microscope. The neutrophil to lymphocyte ratio (N/L ratio) was also calculated. 

### 2.3. Biochemical Parameters

Serum concentrations of glucose, non-esterified fatty acids (NEFA) and total protein (TP) were determined utilizing a clinical system autoanalyzer (Clima MC-15, RAL SA, Barcelona, Spain) and colorimetric reagents (glucose: 11503, Biosystems SA, Barcelona, Spain; NEFA: FA 115, Randox Laboratories Ltd., Crumlin, UK; total protein: GN 46125, Gernon, Barcelona, Spain). The intra- and inter-assay coefficients of variability (CV) were, respectively, 1.2% and 2.7% for glucose, 4.81% and 4.32% for NEFA and 0.88% and 1.23% for TP. The minimum detectable level was 0.0126 mmol/L for glucose, 0.072 mmol/L for NEFA and 2 g/L for TP. Serum protein fractions were separated by electrophoresis using the Hydragel protein (E) K20 kit (Sebia, Lisses, France), according to the manufacturer’s instructions, and results were read with a photodensitometer (Shimadzu CS-9000, Kyoto, Japan).

### 2.4. Acute Phase Proteins and Hormonal Parameters

Serum concentrations of the acute phase proteins haptoglobin (HP) and serum amyloid A (SAA) were measured. HP was determined in microplates, using a colorimetric method based on the haptoglobin–hemoglobin binding (PHASETM Haptoglobin Assay, Tridelta Development Ltd., Maynooth, Ireland), and the concentration of SAA was assessed using a solid phase sandwich ELISA kit (PHASETM Serum Amyloid A Assay, Tridelta Development Ltd., Maynooth, Ireland). The absorbance reading was performed on a microplate reader, Multiskan MS (Labsystems, Helsinki, Finland). The intra- and inter-assay CV were, respectively, 5.3% and 5.7% for HP and 5.0% and 11.4% for SAA. The sensitivity was 0.005 g/L for HP and 0.3 µg/mL for SAA. Serum cortisol was analyzed using a competitive ELISA assay (Salivary Cortisol ELISA SLV-2930, DRG Diagnostics, Marburg, Germany) and the microplates were read with EMS Reader MF V.2.9-0 (Labsystems, Helsinki, Finland). The intra- and inter-assay CV were, respectively, 2.6% and 4.3%, and the sensitivity was 0.25 nmol/L. Corticosterone was extracted from fecal samples, as described by Möstl et al. [11], and analyzed by radioimmunoassay, according to the manufacturer’s instructions of the commercially available kit (ImmuChemTM Double Antibody Corticosterone 125I RIA Kit for rats and mice, ICN Biomedicals Inc. Diagnostic Division, Solon, OH, USA). This assay has been widely used in different species and has been biologically and analytically validated for cattle [12] and biologically validated for lambs [6]. Extraction efficiency, assay accuracy and intra- and inter assay CV were, respectively, 65.5%, 96.3%, 10.1% and 16.8%. Results were expressed as nanograms of metabolite per gram of dry matter (DM). 

### 2.5. Statistical Analysis

Analysis of the hematological and biochemical parameters, acute phase proteins, serum cortisol and FCM was performed by non-parametric tests due to the lack of adjustment respective to normality even after the refinement of the data and the lack of adequate transformations of the variables. The procedure followed was the Friedman test for related samples; when this test was statistically significant, the differences were analyzed using the Wilcoxon signed-rank test for two related samples. In addition, the differences between groups were analyzed for each time sample according to the Mann–Whitney U test (group C vs. group F) or the Kruskall–Wallis test (low, normal and high FCM groups). However, for the acute phase proteins, serum cortisol and FCM, the data analyzed were the changes relative to the value measured at T0 for each animal. The animal weights were analyzed by means of the Generalized Linear Models (GLM) procedure of repeated measures for the study of the evolution of the weight throughout the period or with univariate GLM for the study at each sampling time, with IBM SPSS Statistics V.24. The differences were evaluated post hoc using the Bonferroni test. For the evaluation of clinical signs and lung lesions at the abattoir, the chi-square test was used. For all cases, *p* < 0.05 was required to consider statistically significant differences.

## 3. Results

### 3.1. Hematological Parameters 

There were significant effects of sampling time on RBC, HGB and hematocrit (Table 1). Following the transportation of group F, these parameters significantly decreased relative to the initial sampling (*p* < 0.001 for the three parameters). However, significant differences between the groups were only observed in terms of RBC and hematocrit; specifically, they were higher in the control group at the end of the fattening period (*p* < 0.001 and *p* < 0.05, for RBC and hematocrit, respectively). Regarding the white blood cells (Table 1), the total leukocyte count increased significantly (*p* < 0.001) after transportation to the feedlot. However, for both groups, at the end of the adaptation period, it decreased significantly with regard to T0 (*p* < 0.01, group F, and *p* < 0.001, group C) and elevated again at T3, although it did not recover T0 levels. Neutrophil count evolution was similar to that recorded for leukocytes, but at the end of the fattening period, both groups still maintained significantly lower values than at T0 (*p* < 0.05, group F, and *p* < 0.01, group C). Lymphocyte count significantly decreased after transportation of group F (*p* = 0.01), with no significant differences detected at T2 or T3 compared with T0 for either group. As a result of the neutrophil and lymphocyte counts, the N/L ratio, a stress indicator, was calculated. Thus, following the transport of group F, this parameter was significantly higher (*p* < 0.001) than the initial value recorded. For both groups, N/L was significantly lower at the end of the adaptation period (*p* < 0.001, group F, and *p* < 0.05, group C) and at the end of the fattening (*p* < 0.05, group F, and *p* < 0.001, group C) than at T0. Statistically significant differences between the groups were not found for this parameter, although, at T3, a statistical trend towards a higher value was observed in group F (*p* = 0.079). These modifications in leukocyte profiles occurred within the physiological range, according to Lepherd et al. [13]. 

### 3.2. Biochemical Parameters

Significant effects of sampling time were identified on all biochemical parameters analyzed, but significant differences between the groups were only observed for NEFA, TP and some serum protein fractions (Table 2). Glucose levels did not change significantly throughout the period of the study in the control group, but, in group F, a significant increase was observed at the end of the fattening period compared to T0 (*p* < 0.001). The NEFA value increased significantly after the transportation of group F compared with the initial sampling (*p* < 0.001), but, in both groups, it was significantly lower at the end of the adaptation period (*p* < 0.001, groups F and C) and at the end of the fattening period (*p* < 0.01 in both cases) than at the beginning of the study. However, in group F, a significant increase at T3 was observed relative to T2 (*p* < 0.001), resulting in a significantly higher value than group C at this sampling point (*p* < 0.01). The TP concentration was significantly lower at the end of the adaptation period than at T0 in both groups (*p* < 0.05), but, at the end of fattening, a significant increase was registered in group F in relation to T2 and T0 (*p* < 0.001 in both cases), which resulted in significant differences between the groups at T3 (*p* < 0.05). Regarding the concentration of the different serum protein fractions analyzed, for both groups, significant increases in albumin and α-globulin concentrations were determined at the end of the fattening period compared to T0 (*p* < 0.05 and *p* < 0.001, respectively). In the case of α-globulin, the increase was significantly higher in group F (*p* < 0.001). The concentration of β-globulin significantly decreased in both groups at T2 relative to T0 (*p* < 0.001, group F, and *p* < 0.05, group C), while it increased between T2 and T3 (*p* < 0.001, group F, and *p* < 0.05, group C), with significantly higher levels measured in group F (*p* < 0.05). Regarding the γ-globulin fraction, the concentration significantly increased in group F at T3 relative to T0 and T2 (*p* < 0.001 in both cases), whereas no differences were observed in group C throughout the study, consequently resulting in significantly higher levels at T3 in group F (*p* < 0.001).

### 3.3. Acute Phase Proteins and Hormonal Parameters

**Figure 1:**
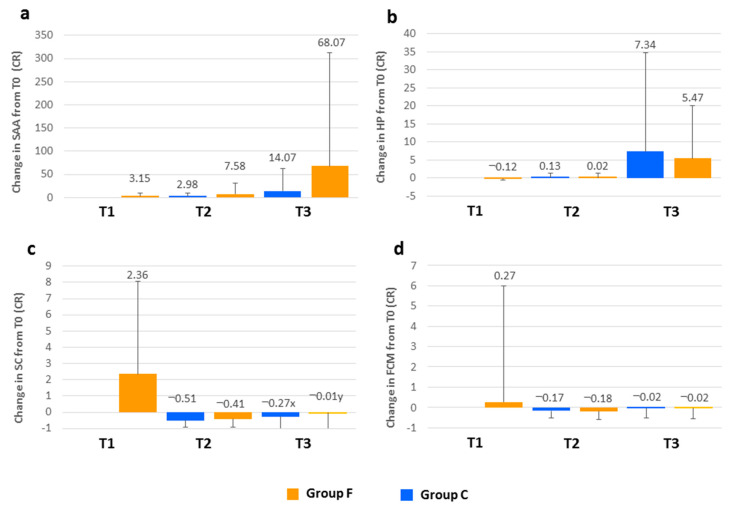
Evolution of (**a**) serum amyloid A (SAA); (**b**) haptoglobin (HP); (**c**) serum cortisol (SC) and (**d**) fecal cortisol metabolites (FCM) throughout the fattening period of lambs fattened in the feedlot (group F) or on the farm of birth (group C). The results at the different sampling times are expressed as a ratio (RC) representing the change relative to the value measured at the initial sampling (T0) for each animal (RC = Tx − T0/T0). T1 sample was collected on arrival at the feedlot, T2 at the end of the adaptation period (two weeks of fattening) and T3 at the end of the fattening period (six weeks of fattening). Different letters (x, y) at the same sampling time indicate statistically significant differences between the groups (*p* < 0.05).

The evolution throughout the period of study of HP, SAA, cortisol and FCM levels in both groups of lambs is presented in Figure 1. The results are expressed as a ratio (CR) indicating the change (increase/decrease) relative to the value measured at T0 for each animal. 

The median value and the 25th and 75th percentile values for HP at T0 for all the lambs in the study were 0.14 (0.10–0.21) g/L. After transportation to the feedlot, the HP concentration in group F was 12% lower than at T0 (CR = −0.12). At the end of the adaptation period, the value registered was similar to the initial value (only 2% higher), but at T3, there was a 5.47-fold increase. In group C, the evolution of HP concentration was very similar and no significant differences between the groups were detected throughout the study period. Analysis of SAA data revealed that, at the beginning of the study, the median value was 1.02 (0.20–6.32) µg/mL. After group F was transported to the feedlot, the value increased 3.15 times, and 7.58 and 68.07-fold increases were determined at the end of the adaptation period and at the end of the fattening period, respectively. For the control group, the evolution of this parameter was analogous, although the increases observed were not as great as those found in the feedlot group. Notwithstanding, statistically significant differences between the groups were not detected. Serum cortisol median value at T0 for the total number of lambs was 21.36 (10.43–56.67) nmol/L. For group F, this value increased 2.36 times after transportation, but, at the end of the adaptation period, serum cortisol decreased substantially in both groups and was 41% (group F) and 51% (group C) lower than at T0. However, a significant increase (*p* < 0.001) was registered between T2 and T3 in group F and at the end of the fattening period, it was very similar to the initial value (1% lower). For group C, the increase was not significant and at the end of the fattening period, serum cortisol was 27% lower than at T0. Differences between the groups were found at T3: the control group demonstrated a greater decrease related to T0 than that determined for group F (*p* < 0.05). For FCM, the median value for the total number of lambs at T0 was 20.12 (13.92–30.37) ng/g DM. The value measured after the transportation of group F to the feedlot was 27% higher than it was on the farm. At the end of the adaptation period, a reduction relative to T0 was observed with both groups (17% and 18% in groups C and F, respectively). Subsequently, the values increased again and at the end of the fattening period, they were very similar to those measured at the beginning of the study (2% lower for both groups). Statistically significant differences were not detected between the groups. 

### 3.4. Final Weight and Health Condition 

**Figure 2:**
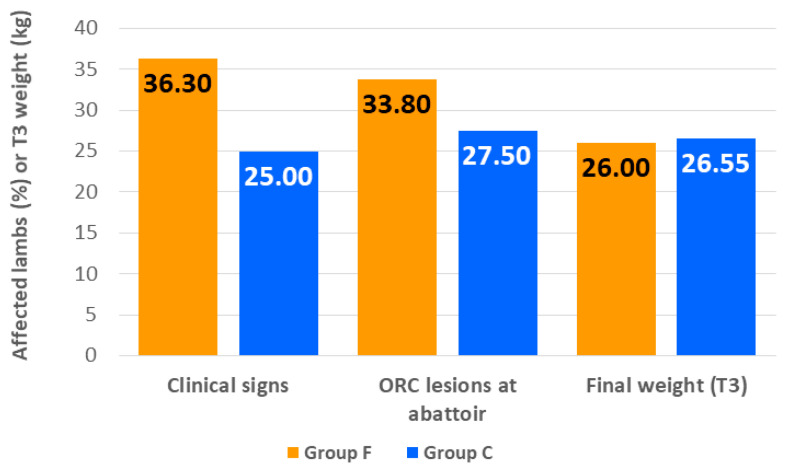
Percentage of lambs with clinical signs of illness throughout the fattening period, percentage of lambs with gross pneumonic lesions compatible with ovine respiratory complex (ORC) identified at the abattoir and average final weight of lambs fattened in the feedlot (Group F) or on the farm of birth (Group C). Statistically significant differences between the groups were not observed for any of the categories (*p* > 0.05).

The average weight of the lambs at the beginning of the study was 13.67 ± 2.082 kg. At the end of the fattening, the lambs fattened on the farm of birth weighed on average 0.55 kg more than those fattened in the feedlot (Figure 2), although differences were not statistically significant (26.55 ± 4.026 kg vs. 26.00 ± 3.451 kg; *p* > 0.05). There were no deaths during the study period but 39 out of the 120 lambs (32.5%) showed clinical signs of illness. The most frequent were apathy and slight fever, non-disease associated (13/39), conjunctivitis (12/39), respiratory signs compatible with ORC (12/39) and diarrhea compatible with coccidiosis (5/39). In all cases, these signs were mild and no lamb needed to be treated. In group F, 29 out of the 80 lambs showed clinical signs and 10/40 in group C. The probability of presenting clinical signs of disease was not affected by the location at which lambs were fattened (Figure 2), although a higher percentage of animals in group F showed clinical signs (36.3% vs. 25.0%, *p* > 0.05). At abattoir, gross pneumonic lesions compatible with ORC were recognized in 38 of the 120 lambs (31.7%). Again, the finishing location did not affect the probability of finding ORC lesions, although a higher proportion of animals fattened in the feedlot developed them (33.8% vs. 27.5%; *p* > 0.05) (Figure 2). 

### 3.5. Individual Responses to Stress, Final Weight and Health Condition

**Figure 3:**
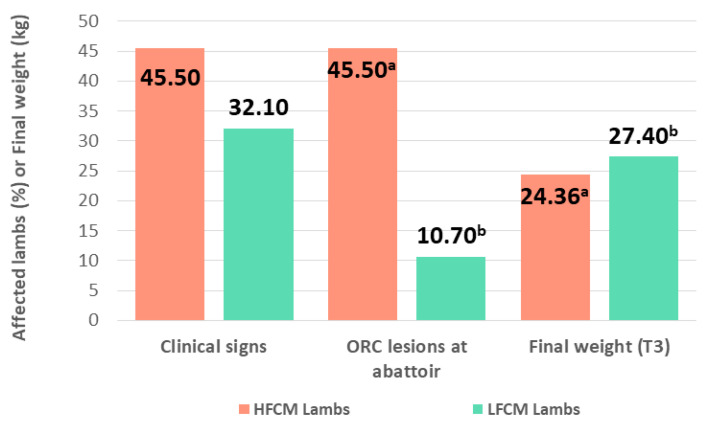
Percentage of lambs with clinical signs of illness throughout the fattening period, percentage of lambs with gross pneumonic lesions compatible with ovine respiratory complex (ORC) identified at the abattoir and average final weight of lambs with concentration of fecal cortisol metabolites at weaning in the highest quartile (HFCM: high fecal cortisol metabolite lambs) and in the lowest quartile (LFCM: low fecal cortisol metabolite lambs). Different letters (a, b) indicate statistically significant differences between the groups (*p* < 0.05).

In order to evaluate whether the level of stress suffered prior to finishing had any influence on the final weight and health of the lambs during the fattening period, the 120 lambs were classified into three categories according to their FCM value (a chronic stress indicator) at T0. Lambs in the highest quartile were identified as high fecal cortisol metabolite lambs (HFCM lambs), lambs in the lowest quartile as LFCM lambs, and the remaining 50% were considered as standard lambs. HFCM lambs showed a higher percentage of clinical signs of illness than LFCM lambs (45.5% vs. 32.1%, *p* > 0.05) and a statistically significant higher proportion of consolidated pneumonic lesions at abattoir (45.5% vs. 10.7%, *p* < 0.01). Furthermore, although the average weight at the beginning of the study was similar in both groups (13.82 kg vs. 13.74 kg, *p* > 0.05), the average final weight was significantly lower in HFCM lambs (24.36 kg vs. 27.40 kg, *p* = 0.001) (Figure 3).

## 4. Discussion

The systemic reaction to stress can affect a wide range of parameters related to the endocrine, metabolic and inflammatory responses. To ascertain the possible impact of stress on the health and the productivity of fattening lambs, this study aimed to investigate the evolution of these parameters throughout the fattening period of lambs transported to feedlots compared with lambs remaining at the farm of origin. 

### 4.1. Hematological Parameters 

One of the most important changes was observed after the transportation to the CC. On arrival, significantly lower RBC, HGB and hematocrit values were detected in the transported lambs compared with values measured on the farm. Previous reports described an increase in these parameters after long trips, attributed to dehydration [5,7]. In our study, lambs were transported by road for one hour, and results are in accordance with Bórnez et al. [4], who reported a slight decrease in these parameters after transportation, of approximately 30 min. Stress induced during handling, loading and unloading could be the cause of an enhancement of free radical production, which could cause erythrocyte lysis [14]. Our results contrast with other reports describing lower values of RBC and hematocrit at the end of the fattening than at the beginning [9,10]. However, in these studies, initial samples were collected after the classification and regrouping of the animals, and possible dehydration during long journeys and the first hours in the feedlot could explain these results. We have also found differences between the groups. At the end of the fattening period, the RBC count and hematocrit were significantly higher in the control group. This could reflect hemolysis enhancement in group F due to oxidative stress, since the serum cortisol level was significantly higher in this group. In our study, transportation to the CC also affected the total leukocyte and neutrophil counts, which significantly increased, whilst lymphocyte numbers significantly decreased, resulting in a significant increase in the N/L ratio. These changes are directly related to an increase in the level of stress hormone in the blood [15], also confirmed in this study. This finding would indicate a stress response associated with transportation, as previously demonstrated in small ruminants by other authors [16,17]. However, a stress response related to abrupt separation from the ewe may also be involved. It is noticeable that, for both groups, the total leukocyte and neutrophil counts and the N/L ratio were significantly higher at T0 than at the end of the adaptation period. Increases in these parameters have also been observed by other authors in abruptly weaned calves [18,19]. It appeared that, at the end of the adaptation period, lambs of both groups had coped with the stress measured at T0, and group F also coped with that associated with transportation, regrouping with other lambs and exposure to a new environment, as significant differences were not found between the groups. At the end of the fattening period, these parameters remained stable compared with T2 or experienced a slight increase, which was generally higher in group F, suggesting a higher stress level in the feedlot group.

### 4.2. Biochemical Parameters

Glucose and NEFA are considered stress indicators in animals, specifically in small ruminants [7,20]. In accordance with this, the higher NEFA level determined for group F at T1 in comparison with T0 could reflect the stress of transportation. In both groups, NEFA values at T0 were significantly higher than at T2, as was observed for the N/L ratio, reinforcing the hypothesis that, at the end of the adaptation period, lambs could overcome the initial stressful situations. The rise in NEFA at T3 in the feedlot group, not observed in the control group, could indicate an underlying stress in the feedlot lambs. This is supported by the fact that the glucose level in group F was significantly higher at the end of fattening than at T0, with no differences throughout the experimental period found in the control lambs. There was a significant effect of sampling time on TP concentration. The statistically significant decrease observed in both groups at the end of the adaptation period could reflect an age-specific variation. Thus, Bórnez et al. [4] reported lower TP levels in light lambs (70 days old) than in suckling lambs. Adaptation to the new diet after weaning could also be an additional factor. Diet based on concentrate, ad libitum provided during the whole fattening period, could explain the significant increase in TP observed in both groups at T3 in relation to T2. Other authors also reported significant increases in TP at the end of the feedlot period, which they attributed to protein supplementation due to increasing requirements for growing lambs [10]. However, TP concentration at T3 was significantly higher in group F than in group C, despite both groups receiving the same type of concentrate. Albumin fraction does not seem to be responsible for this difference, as it increased at the end of the fattening period in both groups and no group differences were observed. However, a significantly higher concentration of the γ-globulin fraction was measured in the feedlot lambs. As both groups were submitted to the same vaccination protocol, this was probably due to exposure to a greater variety of pathogens in the CC as a consequence of housing animals of different origin. The higher concentrations of α- and β- globulin fractions in group F could also be a contributing factor. These fractions include acute phase proteins (APPs) whose concentrations change (increase for the majority) under conditions of inflammation, infection, trauma and stress [21,22]. 

### 4.3. Acute Phase Proteins and Hormonal Parameters

In small ruminants, SAA and HP are considered the major APPs. They are normally present in very low concentrations in healthy goats and sheep but can increase 10–100-fold in response to stimuli [23,24]. Elevated concentrations of these APPs have been reported in response to experimental inflammatory stimulus and under certain infectious conditions [24,25], also in response to transportation stress in ewes [17]. In our study, the median values for HP and SAA serum concentrations at T0 were within the reference range for lambs of similar age [13], although HP was in the upper limit. On arrival at the feedlot, after a 1-h journey by road, serum concentration of SAA was 3.15 times higher. These results contrast with those in ewes reported by Piccione et al. [17]. These authors did not observe significant increases in these APPs before 24-h and 48-h post-transport (for SAA and HP, respectively). Surprisingly, a decrease in HP concentration (12%) was measured after transportation, probably related to the reduction in RBC count, which was also observed at this sampling time. This is probably as a result of the enhancement of free radical production due to the stress of transportation, which could destroy erythrocytes [14] and release hemoglobin, removing HP from circulation. Increased HP concentration in the feedlot group was not observed until the end of the fattening period, six weeks later, suggesting that it is associated with other factors occurring during the fattening period (not only stress-related). The insertion of more sampling times between T1 and T2 would better reveal possible changes in HP concentration related to the stress suffered by the lambs during the initial phases of the study. The increase in SAA concentration occurred earlier and was higher than for HP. In the feedlot group, in addition to the increase observed at T1, an important increase in SAA was measured at the end of the adaptation period (7.58-fold increase), but the greatest increase was revealed at the end of the fattening period (68.07-fold increase). Again, it appeared that other factors occurring during the fattening period could be involved in this response, affecting both groups of animals, as no significant differences between the groups were detected. These factors, apart from multifactorial stressors associated with this period, could include the response to vaccination and also the presence of infection or inflammation. The latter was supported by the fact that lambs with ORC lesions identified at the abattoir registered significantly higher SAA values at T3 than lambs without lesions (data not shown). These results suggest that SAA could be a more useful parameter than HP to provide evidence of an acute phase response during the lamb fattening period. 

Cortisol is the primary corticosteroid released in response to a stressful stimulus and has been widely used as an indicator of stress in animals. Following the transportation of group F to the feedlot, the serum cortisol and the FCM increased relative to T0, reaching the highest value measured throughout the study. This finding is in agreement with previous reports in lambs describing the stressful effects of transport [5,6]. The increase was higher for serum cortisol (2.36-fold increase), whereas FCM rose by 27%. This difference may be attributed to a time delay between increased serum cortisol levels and their reflection in the excreted FCM [26]. It must be highlighted that, without considering the post-transport sampling, both groups of lambs showed the highest level for both parameters at T0, consistent with other stress indicators measured in this study (N/L ratio and NEFA), corroborating a high stress level at this time. In the case of serum cortisol, it would be related to the emotional stress due to abrupt separation from the ewe. Other authors have also reported high cortisol levels in weaned lambs [4,27,28]. This could have induced some effect on the values measured after transportation, although Napolitano et al. [27] observed that serum cortisol returned to basal levels 45 min after lambs were removed from their dams. However, the high level of FCM found at T0 would reflect the effect of stressors experienced by lambs prior to this time point, such as reduced access to milk in the previous days, as the amount of feed provided to the ewes was lower during the last days of lactation in order to facilitate the drying of the udder. In fecal samples, circulating hormone levels are integrated over a time period and represent the cumulative secretion of the hormone, unlike serum cortisol that is affected by short episodic fluctuations and the pulsatile hormone secretion [26]. For this reason, FCM is considered an indicator of chronic stress. The serum cortisol and FCM levels decreased at the end of the adaptation period for both groups. Again, probably for the same reason, the decrease was higher for serum cortisol (51% and 41%) than for FCM (17% and 18%) in groups C and F, respectively. Statistically significant differences between the groups were not observed at this sampling time. It would appear that, at the end of the adaptation period, group F had coped with the initial stress and also with that associated with transportation, exposure to a new environment and regrouping with other lambs. The same pattern was observed in relation to the N/L ratio and NEFA, reinforcing this hypothesis. As demonstrated by the FCM and serum cortisol values, the stress level increased again in both groups at the end of the fattening period. The significant differences found in serum cortisol level between the groups suggest that the feedlot group experienced higher stress levels than the control group. The presence of underlying multifactorial stress factors associated with the feedlot could be involved in these differences. 

### 4.4. Health Condition and Final Weight

A study investigating lamb feedlot production in Spain, involving 1243 lambs, determined that the clinical signs of ORC are some of the most prevalent (8.83%) [1]. Our results agree with this finding, as respiratory clinical signs compatible with ORC were present in 12 out of the 120 lambs. The percentage of ORC lesions found in our study (31.7%) was also analogous with the 29.9% reported by Luzón [29] for lambs of the same breed and production system. In addition, the final weights were those expected for the Rasa Aragonesa breed [30]. Relevant information derived from our study demonstrates that the location at which lambs were fattened did not appear to affect the health or the final weight of the animals. Lambs fattened in the feedlot presented a higher percentage of any form of clinical signs and ORC lesions than those fed on the farm, with their final weight being lower. Nonetheless, none of these differences were statistically significant. However, animals from group F endured, as a result of transportation, the highest level of stress registered in the study, as reflected by several of the stress indicators evaluated (N/L ratio, NEFA, serum cortisol and FCM). By the end of the adaptation period, these parameters had decreased and values equivalent those of lambs in the control group were determined. Nevertheless, at the end of the fattening period, all of them rose and NEFA and serum cortisol values were significantly higher than in the control group. These results seem to indicate that lambs fattened in the feedlot could experience higher stress levels than those fattened on the farm of origin. Notwithstanding, these differences did not appear to significantly affect the health or the final weight of the lambs. It is known that stress responses can vary in individual animals and the existence of different stress reaction patterns within a population has been demonstrated [31]. The individual variability obtained for the stress indicators evaluated in this study is in agreement with these findings. Without considering the post-transport sampling, for both groups of lambs, the highest levels of stress indicators were measured at the beginning of the study, at the time of weaning. For this reason, the influence of the intensity of the stress response at this time on the health and the final weight of the lambs was evaluated. FCM concentration was used, as it is an indicator of chronic stress. It would reflect the effect of stressful situations experienced by lambs prior to T0 and it is less responsive to short term handling stressors. In this way, independent of the location at which lambs were fattened, HFCM lambs developed a higher percentage of clinical signs of illness during the fattening period, a significantly higher percentage of ORC lesions and their average final weight was significantly lower than LFCM lambs. These results appear to indicate that response to stressors experienced by lambs prior to finishing could affect their further development, and FCM at the time of weaning could be used as a predictor of health and production outcomes during the subsequent fattening period. Taking these considerations into account, we suggest that changes in management practices should be investigated in order to reduce the stress level experienced by the lambs in the previous days to weaning. This could prove to be a useful tool to improve the health and productivity of the lambs during the fattening period, contributing to the sustainability of the ovine production system and helping to reduce the use of antibiotics.

## 5. Conclusions

The differences observed in the stress markers analyzed in this study suggest that lambs fattened in the feedlot could have experienced higher stress levels than those fattened on the farm of origin. However, the location at which lambs were fattened did not affect their health or their final weight. Apart from the post-transport sampling, the highest stress level in both groups of lambs was measured at the beginning of the study, at the time of weaning. FCM concentration at this time seems to be relevant for the health and productive performance of the lambs during the fattening period, regardless of finishing location.

## Figures and Tables

**Table 1:** Hematological parameters (median value and 25th and 75th percentile values) of fattening lambs at different blood sampling times throughout the fattening period.

Variable ^†^			Sampling Time ^‡^		
	Group ^§^	T0	T1	T2	T3
RBC (10^12^ cells/L)	C	10.77 (10.12–11.07)		10.48 (10.03–11.29)	11.02^x^ (10.30–11.75)
	F	10.24^a^ (9.19–10.74)	9.81^b^ (8.51–10.39)	10.33^a^ (9.63–11.10)	10.23^a,y^ (9.81–11.05)
HGB (g/L)	C	104.50^a^ (99.00–111.50)		107.50^b^(103.00–116.00)	110.00^ab^ (102.25–116.75)
	F	105.50^a^ (97.00–114.00)	101.00^b^ (91.25–106.75)	110.00^c^ (103.00–117.25)	109.00^ac^ (99.00–115.50)
Hematocrit (%)	C	33.50^ab^ (32.00–35.36)		33.75^a^ (30.00–35.00)	35.25^b,x^ (31.13–36.86)
	F	33.00^a^ (30.00–35.00)	31.00^b^ (28.50–33.00)	32.45^a^ (30.50–35.50)	34.00^a,y^ (30.38–36.00)
WBC (10^9^ cells/L)	C	7.20^a^ (5.65–8.50)		6.05^b^ (4.73–7.63)	6.40^ab^ (5.70–7.45)
	F	7.00^a^ (5.75–8.05)	8.20^b^ (6.43–9.38)	6.10^c^ (5.35–7.40)	6.50^ac^ (5.30–7.60)
Neutrophils (10^9^ cells/L)	C	2.76^a^ (2.17–4.00)		2.28^b^ (1.46–2.93)	2.03^b^ (1.67–2.61)
	F	2.80^a^ (2.12–3.91)	4.20^b^ (3.02–5.39)	2.04^c^ (1.70–2.59)	2.38^c^ (1.65–3.41)
Lymphocytes (10^9^ cells/L)	C	3.40 (2.67–4.59)		3.12 (2.55–4.84)	3.90 (3.12–4.47)
	F	3.46^a^ (2.83–4.21)	3.10^b^ (2.58–3.96)	3.48^a^ (2.77–4.29)	3.54^a^ (2.98–4.17)
N/L ratio	C	0.81^a^ (0.59–1.32)		0.66^b^ (0.47–0.87)	0.58^b^ (0.40–0.78)
	F	0.83^a^ (0.58–1.20)	1.35^b^ (0.88–1.77)	0.67^c^ (0.43–0.82)	0.71^c^ (0.41–1.02)

^†^ RBC = red blood cells; HGB = hemoglobin; WBC = white blood cells; N/L ratio = neutrophil to lymphocyte ratio. ^‡^ T0 = at weaning on the farm of origin; T1 = on arrival at the feedlot after road transportation; T2 = at the end of the adaptation period (2 weeks); T3 = at the end of the fattening period (6 weeks). ^§^ C = group of lambs fattened on the farm of origin; F= group of lambs fattened in the feedlot. ^a–d^ Different letters within a row indicate significant differences (*p* < 0.05) between the different sampling times. ^x,y^ Different letters within columns indicate significant differences (*p* < 0.05) between groups C and F at the same sampling time.

**Table 2:** Biochemical parameters (median value and 25th and 75th percentile values) of fattening lambs at different blood sampling times throughout the fattening period.

Variable ^†^		Sampling Time ^‡^			
	Group ^§^	T0	T1	T2	T3
Glucose (mmol/L)	C	5.12 (3.99–5.73)		5.19 (4.35–5.71)	4.97 (4.52–5.69)
	F	4.76^a^ (4.35–5.27)	4.89^ab^ (4.18–5.63)	4.91^a^ (4.40–5.58)	5.22^b^ (4.86–5.65)
NEFA (mmol/L)	C	0.24^a^ (0.13–0.34)		0.12^b^(0.10–0.17)	0.14^b,x^ (0.10–0.18)
	F	0.28^a^ (0.21–0.68)	0.43^b^ (0.31–0.80)	0.10^c^ (0.07–0.14)	0.23^d,y^ (0.13.0.37)
Total protein (g/L)	C	54.85^a^ (49.58–58.15)		52.35^b^ (48.43–56.73)	56.30^a,x^ (50.38–59.80)
	F	54.15^a^ (51.25–58.63)	53.50^ab^ (51.00–57.00)	53.25^b^ (51.10–56.48)	57.15^c,x^ (54.08–60.50)
Albumin (g/L)	C	35.46^a^ (31.54–38.95)		34.14^a^ (31.03–37.63)	37.64^b^ (31.82–40.20)
	F	34.57^a^ (31.73–34.37)	34.37^a^ (32.33–37.13)	34.84^a^ (32.36–36.53)	35.41^b^ (34.21–37.68)
α-globulins (g/L)	C	2.45^a^ (2.27–2.76)		2.64^b^ (2.25–2.92)	2.66^b,x^ (2.41–3.05)
	F	2.50^a^ (2.22–2.70)	2.45^a^ (2.22–2.71)	2.67^b^ (2.50–2.99)	2.98^c,y^ (2.72–3.24)
β-globulins (g/L)	C	11.25^a^ (10.42–12.38)		10.66^b^ (9.83–11.44)	10.92^a,x^ (10.17–11.90)
	F	11.73^a^ (10.85–12.88)	11.41^b^ (10.19–12.47)	10.52^c^ (9.78–11.08)	11.65^ab,y^ (10.68–12.53)
γ-globulins (g/L)	C	4.91 (4.22–6.07)		4.91 (4.19–5.95)	5.01^x^ (4.46–6.13)
	F	5.18^a^ (4.20–6.44)	5.02^a^ (4.15–6.19)	5.05^b^ (4.61–6.25)	6.65^c,x^ (0.41–1.02)

^†^ NEFA = non-esterified fatty acids. ^‡^ T0 = at weaning on the farm of origin; T1 = on arrival at the feedlot after road transportation; T2 = at the end of the adaptation period (2 weeks); T3 = at the end of the fattening period (6 weeks). ^§^ C = group of lambs fattened on the farm of origin; F = group of lambs fattened in the feedlot. ^a,b,c,d^ Different letters within a row indicate significant differences (*p* < 0.05) between the different sampling times. ^x,y^ Different letters within columns indicate significant differences (*p* < 0.05) between groups C and F at the same sampling time.
